# Rationalization of Solvent-Mediated Enthalpy–Entropy Compensation with the Gibbs–Helmholtz Equation

**DOI:** 10.1007/s10930-026-10358-0

**Published:** 2026-10-30

**Authors:** David J. Scott, Donald J. Winzor

**Affiliations:** 1 School of Biosciences, University of Nottingham, Sutton Bonington Campus, Loughborough, LE12 5RD UK; 2 School of Chemistry and Molecular Biosciences, University of Queensland, Brisbane, QLD 4072 Australia

**Keywords:** Protein-ligand interactions, Enthalpy–entropy compensation, Gibbs–Helmoltz equation, van’t Hoff isochore, Calorimetry

## Abstract

This review examines the effect of solvent involvement in protein-ligand interactions on the extent of conformity with the Gibbs–Helmholtz equation, which was based on assumed inertness of the solvent—an assumption that rendered possible the evaluation of Δ*H*^*o*^ and *T*Δ*S*^*o*^ by employing the integrated form of the van’t Hoff isochore. Conformity with the Gibbs–Helmholtz equation is retained in situations where there is temperature dependence of the calorimetrically measured standard enthalpy change despite no change in standard free energy change (ΔΔ*G*^*o*^ ≈ 0). Before the advent of isothermal titration calorimetry this situation was taken to signify that Δ*H*^*o*^ = 0 rather than *(ΔH*^*o*^)_vH_ = 0, whereupon (Δ*G*^*o*^)_vH_ = (*T*Δ*S*^*o*^)_vH_. In isothermal titration calorimetry the measured standard enthalpy change (Δ*H*^*o*^)_cal_ is a thermodynamic parameter for the whole system, whereupon values of (*T*Δ*S*^*o*^)_cal_ calculated as (Δ*H*^*o*^)_cal_–(Δ*G*^*o*^)_vH_ include the van’t Hoff enthalpy contribution (Δ*H*^*o*^)_vH_. A sufficiently large magnitude for that van’t Hoff contribution leads to a Gibbs–Helmholtz plot with a slope greater than unity. A similar situation arises in systems exhibiting a slight but significant dependence of (Δ*G*^*o*^)_vH_ upon (Δ*H*^*o*^)_cal_ —examples of systems exhibiting substantial rather than extreme enthalpy–entropy compensation.

## Introduction

Extreme enthalpy–entropy compensation is a term that was introduced to describe situations where the interactions of proteins with charged ligands [1–4] and their apolar counterparts [5–7] give rise to a significant standard enthalpy change (Δ*H*^*o*^) that is countered by an entropy energy contribution (*T*Δ*S*^*o*^) to the extent that there is effectively no change in standard free energy (ΔΔ*G*^*o*^ ≈ 0). Although the phenomenon has been attributed to compensatory structural entropy changes [8–11], a more likely explanation entails the involvement of changes in water structure [12–15]. An obvious attraction of the latter explanation is that any gain in the enthalpic contribution (Δ*H*^*o*^) to the standard free energy (Δ*G*^*o*^) from hydrogen bond formation involving water hydroxyls is necessarily accompanied by a concomitant loss in the entropy contribution (*T*Δ*S*^*o*^) because of decreased randomness of a more ordered water structure. Failure to consider this possibility reflected the reluctance of many researchers to regard the solvent as part of the thermodynamic system under study. Such implicitly assumed inertness of the aqueous solvent has led to observed temperature independence of Δ*G*^o^ values being considered to signify that Δ*H*^*o*^ = 0 rather than (Δ*H*^*o*^ – *T*Δ*S*^*o*^) = 0 [16, 17]. The goal of this communication is to rationalize the consequences of solvent involvement on the application of the Gibbs–Helmholtz equation.

## Consideration of Specific Protein-Ligand Interactions

The practice of disregarding the solvent as part of the thermodynamic system under investigation was deeply entrenched in physicochemical as well as biochemical investigations for a long time. However, the emergence of systems with extreme enthalpy–entropy compensation (standard free energy change insensitive to change in temperature) introduces confusion into their interpretation in terms of the Gibbs–Helmholtz equation.

Although the Gibbs–Helmholtz equation is routinely regarded as a thermodynamic expression, its interpretation of the difference between the energies of the initial and final states in molecular terms of an equilibrium constant, the values of the standard enthalpy, standard free energy and standard enthalpy energy contributions become model-dependent. The expressions for a two-component system, A + B ⇌ C governed by association equilibrium constant *K*, dispersed in an inert solvent are

1 $$\:(\triangle{G}^{o}{)}_{vH}=-RT\mathrm{l}\mathrm{n}\:K$$

2 $$\:( \triangle{{G}^{o})}_{vH}=( \triangle{H}^{o}{)}_{\mathrm{v}\mathrm{H}}-(T \triangle{S}^{o}{)}_{vH}$$

3 $$\:\mathrm{ln}K=\frac{( \triangle{S}^{o}{)}_{\mathrm{v}\mathrm{H}}}{R}-\frac{( \triangle{H}^{o}{)}_{\mathrm{v}\mathrm{H}}}{RT}$$

where the vH subscript is used to emphasize their derivation for a system conforming with description in term of the van’t Hoff reaction isochore. At the time of their development the inherently assumed solvent inertness was a reasonable approximation because of the large magnitudes of the association constants for the systems being investigated. However, over the years the same approach has been applied to characterize much weaker interactions, where energy contributions arising from the hydrogen-bonding ability of water may well need to be considered.

In that regard the advent in 1989 of isothermal titration calorimetry [18] at last afforded a direct means of determining the enthalpy change for the whole system, ($\:( \triangle{H}^{o}{)}_{\mathrm{c}\mathrm{a}\mathrm{l}}$), as well as the binding constant *K* and hence $\:(\triangle{G}^{o}{)}_{vH}$. An experimental estimate of the entropic energy contribution was then obtained as.

4 $$\:(T\triangle{S}^{o}{)}_{\mathrm{c}\mathrm{a}\mathrm{l}}=(\triangle{H}^{o}{)}_{\mathrm{c}\mathrm{a}\mathrm{l}}-(\triangle{G}^{o}{)}_{\mathrm{v}\mathrm{H}}$$

which, on incorporating Eq. (2), becomes.

5 $$\:(T\triangle{S}^{o}{)}_{\mathrm{c}\mathrm{a}\mathrm{l}}=(\triangle{H}^{o}{)}_{\mathrm{c}\mathrm{a}\mathrm{l}}-(\triangle{H}^{o}{)}_{\mathrm{v}\mathrm{H}}+(T\triangle{S}^{o}{)}_{vH}$$

The calorimetric entropic energy contribution thus incorporates the difference between $\:(\triangle{H}^{o}{)}_{\mathrm{c}\mathrm{a}\mathrm{l}}$ and $\:(\triangle{H}^{o}{)}_{\mathrm{v}\mathrm{H}}$ as well as the van’t Hoff entropic energy contribution $\:(T\triangle{S}^{o}{)}_{\mathrm{v}\mathrm{H}}$.

An ability to determine $$\:(\triangle{H}^{o}{)}_{\mathrm{c}\mathrm{a}\mathrm{l}}$$ has thus allowed direct access to $$\:(T\triangle{S}^{o}{)}_{\mathrm{c}\mathrm{a}\mathrm{l}}$$, the final energy parameter required for description of a given protein-ligand system in terms of the Gibbs–Helmholtz equation, thereby eliminating the need for temperature-dependence studies to evaluate $$\:( \triangle{H}^{o}{)}_{\mathrm{v}\mathrm{H}}$$ via Eq. (3). This development has also broadened the scope of thermodynamic studies for the detection of enthalpy–entropy compensation by incorporating studies in which the substitution of a range of related ligands is used to effect a change in $$\:(\triangle{H}^{o}{)}_{\mathrm{c}\mathrm{a}\mathrm{l}}$$ at a single temperature. In that regard there is enthalpy–entropy compensation whenever $$\:(\triangle{G}^{o}{)}_{vH}\neq$$$$\:(\triangle{H}^{o}{)}_{\mathrm{c}\mathrm{a}\mathrm{l}}$$; but the emphasis in this investigation is on protein-ligand interactions where $$\:(\triangle{G}^{o}{)}_{vH}$$ is essentially independent of $$\:( \triangle{H}^{o}{)}_{\mathrm{c}\mathrm{a}\mathrm{l}}$$—a situation more definitively defines as extreme enthalpy–entropy compensation.        

We now take advantage of these quantitative expressions by their application to results from several calorimetric studies on specific protein-ligand interactions in which the existence of solvent involvement has been established by Gibbs–Helmholtz analysis.

### Interaction of Ribonuclease A with Cytidine 2’-Monophosphate

**Fig. 1:**
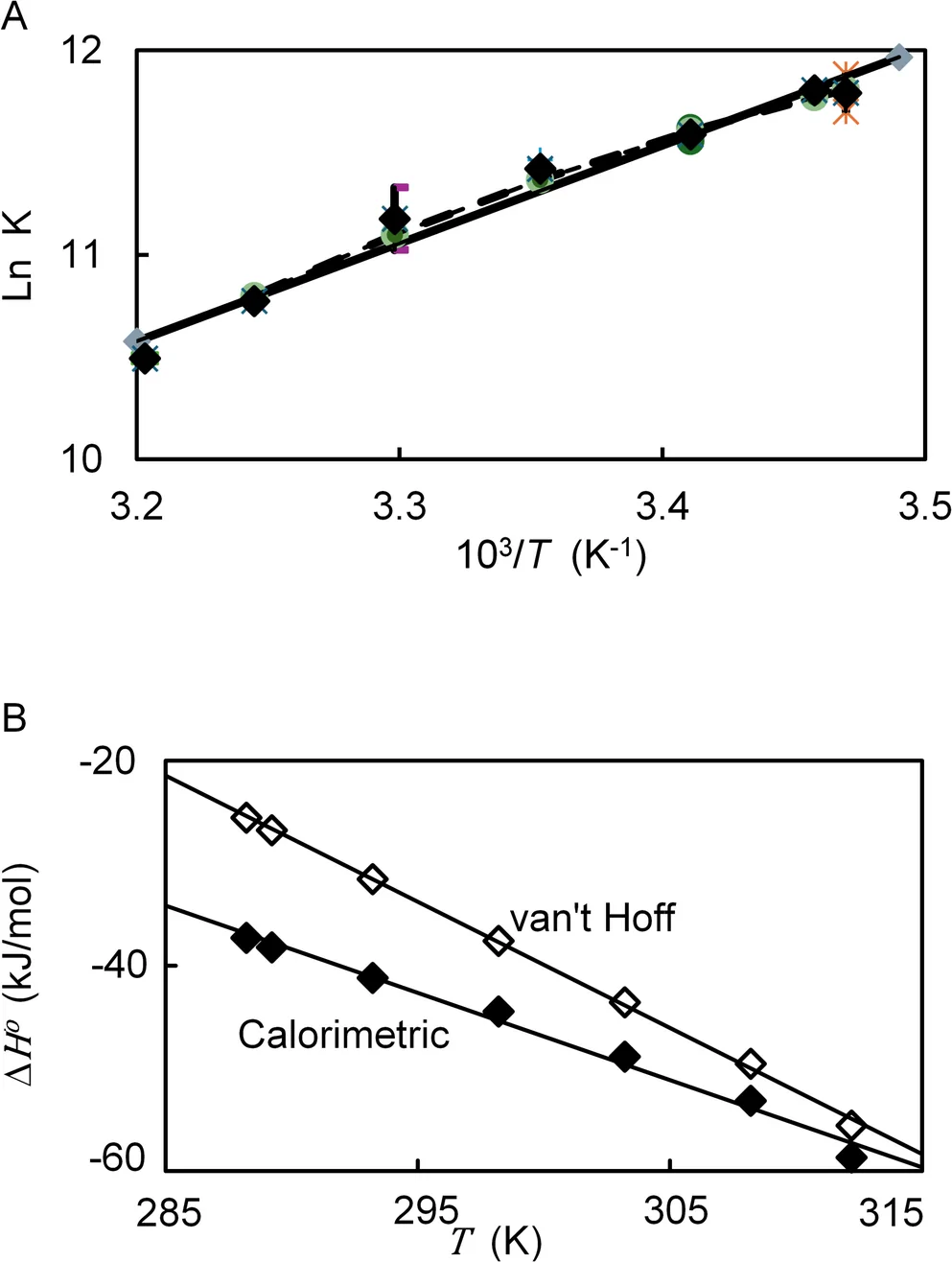
Calorimetric studies of the interaction between 2’-cytidine monophosphate and ribonuclease A. **A** Classical van’t Hoff plot of results (●) according to Eq. (3) (^_____^) and the modified van’t Hoff analysis (– – –) according to Eq. (6): an error bar is inserted to denote the uncertainty in one experiment. **B** Temperature dependence of the standard enthalpy change obtained by the modified analysis (◇) and by direct calorimetric measurement (◆). (Data taken from Table 1 of [19].)

In keeping with tradition, association constants from an isothermal calorimetry study of the interaction between ribonuclease and cytidine 2’-monophosphate [19] were initially subjected to analysis by means of the integrated form of the van’t Hoff isochore, Eq. (3), which yielded an estimate of − 39.6 kJ/mol for $\:(\triangle{H}^{o}{)}_{\mathrm{v}\mathrm{H}}$from the slope of the essentially linear dependence of ln *K* upon 1/*T* (solid line in Fig. 1A). Because that finding conflicted with its temperature-dependent calorimetric counterpart (Fig. 1B), an attempt was made to introduce temperature dependence of Δ*H*^*o*^ via a more general form of the integrated van’t Hoff isochore [20],

6 $$\:\mathrm{ln}\frac{K}{{K}_{o}}=\frac{( \triangle{H}^{o}{)}_{\mathrm{v}\mathrm{H}}-{T}_{o}\triangle{C}_{p}}{R}\left(\frac{1}{{T}_{o}}-\frac{1}{T}\right)+\frac{ \triangle{C}_{p}}{R}\left(\mathrm{l}\mathrm{n}\frac{T}{{T}_{o}}\right)$$

which describes the temperature dependence of the association constant in terms of its value (*K*^*o*^) and that of the van’t Hoff enthalpy, $\:( \triangle{H}^{o}{)}_{\mathrm{v}\mathrm{H}}$, at selected temperature *T*_*o*_ for a system with temperature-independent heat capacity change (Δ*C*_p_). Nonlinear regression analysis of the temperature dependence of *K* in terms of Eq. (6) led to the curvilinear variation of ln *K* shown as the dashed line in Fig. 1A. Although such action predicted negative temperature dependence of $\:( \triangle{H}^{o}{)}_{\mathrm{v}\mathrm{H}}$, the extent was an underestimate of$\:\:( \triangle{H}^{o}{)}_{\mathrm{c}\mathrm{a}\mathrm{l}}$, its experimental and unequivocal calorimetric counterpart (Fig. 1B). It was therefore conceded that the discrepancies between calorimetric and van’t Hoff descriptions of the energetics must reflect the existence of additional temperature-dependent changes in equilibria controlling the interactions of buffer or solution components, including water, with one or more of the components of the complex [19].

**Fig. 2:**
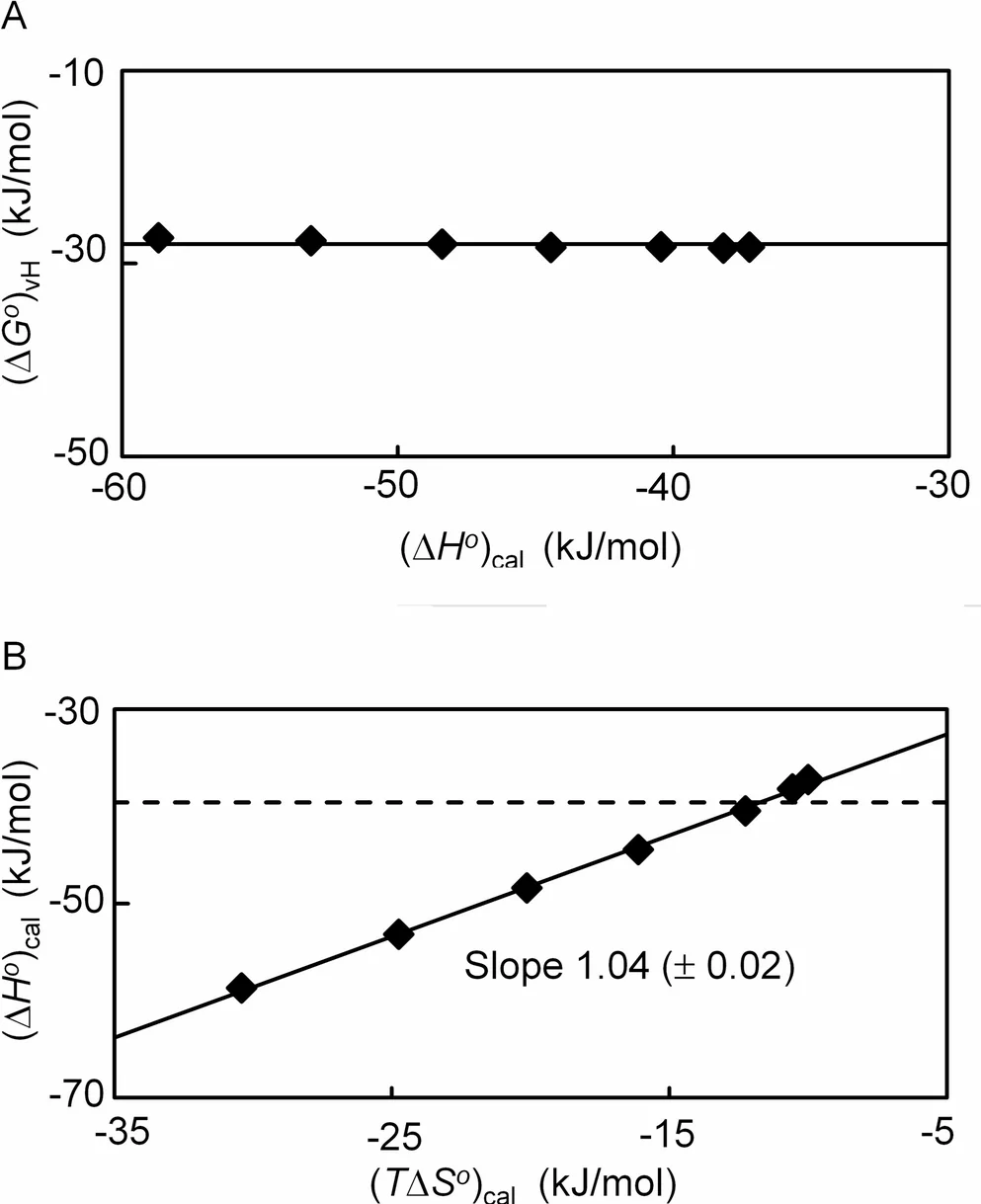
Reanalysis of the above results for interaction between 2’-cytidine monophosphate and ribonuclease A in terms of the enthalpy–entropy compensation phenomenon. **A** Demonstration of the temperature independence of the experimentally measured standard free energy change (Δ $\:{G}^{o}{)}_{\mathrm{v}\mathrm{H}}$ upon (Δ*H*^*o*^)_cal_. **B** The Gibbs–Helmholtz plot (^______^) based on the enthalpic contribution calculated from Eq. (5): the broken line reflects the value of (Δ*H*^*o*^)_vH_ calculated from Eq. (3)

In fact, the results from that calorimetric study by the Sturtevant group [19] have provided a good illustration of extreme enthalpy–entropy compensation. This is evident from Fig. 2A, which demonstrates the independence of $\:( \triangle{G}^{o}{)}_{\mathrm{v}\mathrm{H}}$ upon $\:( \triangle{H}^{o}{)}_{\mathrm{c}\mathrm{a}\mathrm{l}}$. Analysis of the results in terms of Eq. (4) is summarized in Fig. 2B, where the slope of the Gibbs–Helmholtz plot is slightly greater than unity—a manifestation of the incorporation of $\:( \triangle{H}^{o}{)}_{\mathrm{v}\mathrm{H}}$ into the magnitude of (*T*Δ*S*^*o*^)_cal_, Eq. (5). This behavior contrasts markedly with the temperature independence of $\:(\triangle{H}^{o}{)}_{\mathrm{v}\mathrm{H}}$ (– – –) predicted for an interaction with no solvent interaction (Eq. (2)).

**Fig. 3:**
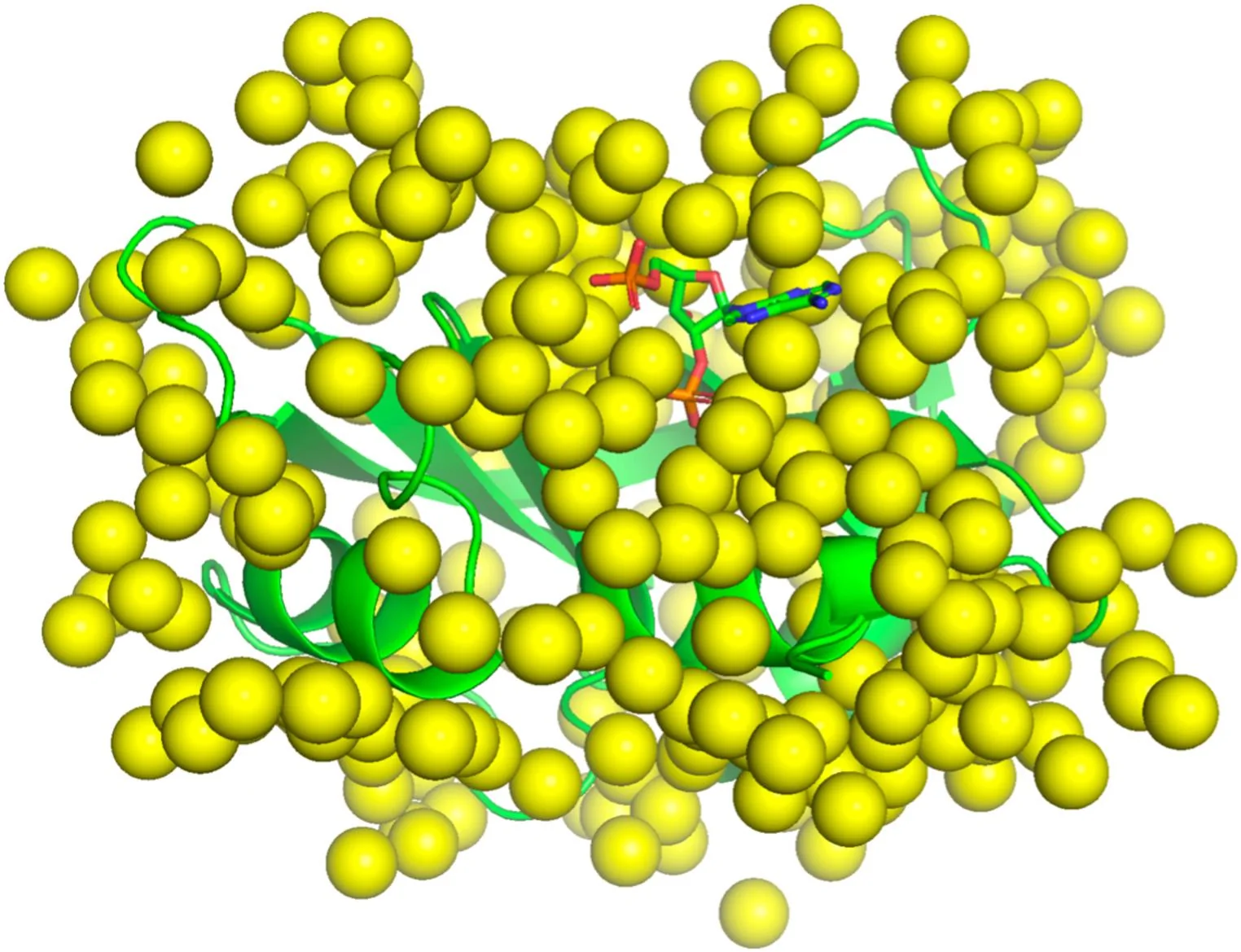
Three-dimensional crystal structure of the complex between adenine-2’5’-diphosphate and ribonuclease [21] showing the presence of water molecules (yellow) in the vicinity of the polypeptide chain (green) as well as the active site. (Structure constructed from coordinate listed in the Brookhaven Protein Data Bank.)

The main features to emerge from Fig. 2 are (i) the independence $\:( \triangle{G}^{o}{)}_{\mathrm{v}\mathrm{H}}$ upon $\:( \triangle{H}^{o}{)}_{\mathrm{c}\mathrm{a}\mathrm{l}}$ (Fig. 2A) and the difference between the temperature dependence of $\:( \triangle{H}^{o}{)}_{\mathrm{c}\mathrm{a}\mathrm{l}}$ (^_____^) and the inherently assumed constancy of $\:( \triangle{H}^{o}{)}_{\mathrm{v}\mathrm{H}}$ (– – –) in its evaluation via Eq. ((3) (Fig. 2B). Both are consistent with the existence of solvent interaction in the binding of ribonuclease A with adenosine-2’5’-diphosphate. Evidence for the existence of hydrogen-bonded and other water molecules in complexes of ribonuclease A with adenosine-2’5’-diphosphate had already been revealed in high-resolution crystal structures [21], This is highlighted in the three-dimensional structure (Fig. 3) constructed from coordinates listed in the Brookhaven Protein Data Bank. Water molecules (highlighted in yellow) are clearly associated with the protein polypeptide chain (green) in regions other than the binding site, presumably those responsible for the empirical correlation between hydrophobic energy and aqueous cavity surface area [14]. Of greater relevance in the present context is the cluster of water molecules in the hydrophobic binding site, where 19 hydrogen-bonded water molecules have been detected: Fig. 2B and C of the Leonidas article [21] identify the locations of those hydrogen-bonded water molecules.

### Interaction of Dipeptide Binding Protein DppA with Alanine Dipeptides

**Fig. 4:**
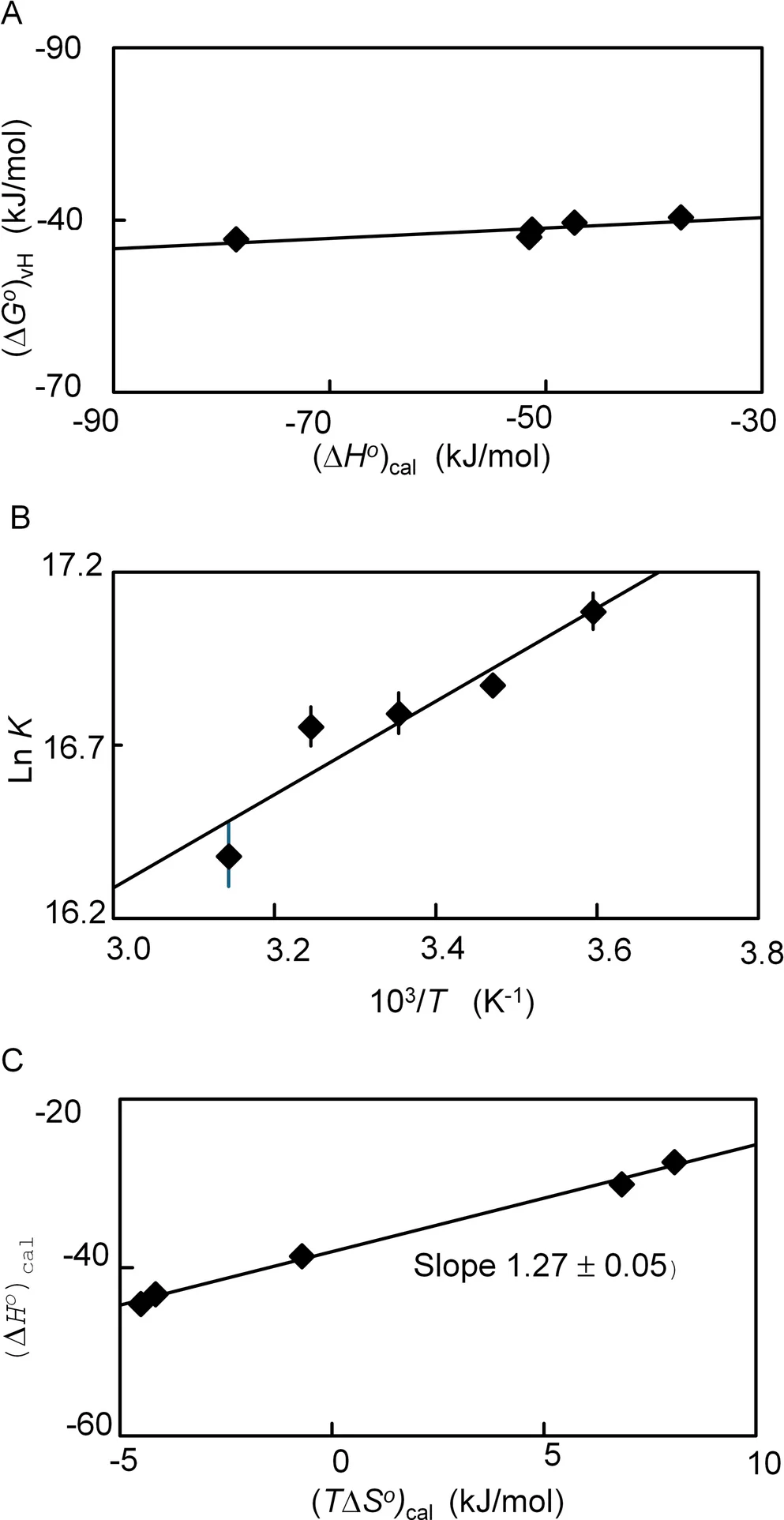
Interaction of the dipeptide FA with DppA. **A** Temperature dependence of the experimentally measured standard free energy change, (Δ$\:{G}^{o}$)_vH_ upon its calorimetrically measured enthalpic counterpart, (Δ*H*^*o*^)_*cal*_. **B**, Classical van’t Hoff plot for the determination of (Δ*H*^*o*^)_vH_. **C** Gibbs–Helmholtz plot of results with (*T*Δ*S*o)_cal._calculated from Eq. (5). (Data taken from [22]

Results from an isothermal titration calorimetry study [22] of interactions between the dipeptide binding protein DppA and three alanine dipeptides, AA, AF and FA, were also symptomatic of extreme enthalpy–entropy compensation in that a pronounced temperature dependence was observed for the standard enthalpy change, $\:( \triangle{H}^{o}{)}_{\mathrm{c}\mathrm{a}\mathrm{l}}$, despite only minor change in standard free energy. Structural changes leading to confinement of the binding site within a hydrophobic environment in a DppA-dipeptide complex [22, 23], where their 2.8 Å resolution precludes the definitive detection of water molecules within that environment. Whereas $\: \triangle{G}^{o}$ for the first two dipeptides could be deemed effectively constant, as in Fig. 2A, the corresponding temperature dependence for FA was significant (Fig. 4A). We now need to ascertain the consequences of this temperature dependence on the present rationalization of extreme enthalpy–entropy compensation in terms of the Gibbs–Helmholtz equation.

The first step entails use of Van’t Hoff isochore, Eq. (2), to evaluate $\:(\triangle{H}^{o}{)}_{\mathrm{v}\mathrm{H}}$ from the slope of the dependence of ln *K* upon 1/*T* (Fig. 4B): a value of − 3.1 kJ/mol is thereby obtained. It therefore follows that the magnitude of $\: \triangle{G}^{o}$ is dominated by a positive $\:(T\triangle{S}^{o}{)}_{\mathrm{v}\mathrm{H}}$ contribution that reflects a decreased alignment of water molecules within the protein binding site as the result of charge neutralization effected by complex formation, a phenomenon predicted by Kauzman [24]. Furthermore, reference to Eq. (5) establishes that the small magnitude of $\:( \triangle{H}^{o}{)}_{\mathrm{v}\mathrm{H}}$ shows that its inclusion in $\:(T\triangle{S}^{o}{)}_{\mathrm{c}\mathrm{a}\mathrm{l}}$ renders reasonable the approximation that $\:(T\triangle{S}^{o}{)}_{\mathrm{c}\mathrm{a}\mathrm{l}}$ ≈ $\:(T \triangle{S}^{o}{)}_{\mathrm{v}\mathrm{H}}.$ A Gibbs–Helmholtz plot of the present results is presented in Fig. 4C, where the deviation of the slope from unity could well reflect the variation of $\:(\triangle{G}^{o}{)}_{\mathrm{v}\mathrm{H}}$with $\:( \triangle{H}^{o}{)}_{\mathrm{v}\mathrm{H}}$ (Fig. 4A). To that end Fig. 4B is effectively a plot of the results according to Eq. (2); and the magnitude of − 38 (± 0.3) kJ/mol inferred for $\:(\triangle{G}^{o}{)}_{\mathrm{v}\mathrm{H}}$ from the ordinate intercept of Fig. 4B is indistinguishable from the mean value of the standard free energy changes for the data set – the value that gives rise to a Gibbs–Helmholtz plot with a slope of unity. Those findings are considered to justify attributing Gibbs–Helmholtz plots with slopes markedly greater than unity to slight but significant variation of $\:(\triangle{G}^{o}{)}_{\mathrm{v}\mathrm{H}}$ with $\:(\triangle{H}^{o}{)}_{\mathrm{c}\mathrm{a}\mathrm{l}}$.

## Calorimetric Studies at a Single Temperature

In the two systems considered thus far the calorimetric studies have been conducted at a range of temperatures to allow advantage to be taken of the van’t Hoff reaction isochore to achieve the breakdown of $\:(\triangle{G}^{o}{)}_{\mathrm{v}\mathrm{H}}$ into $\:( \triangle{H}^{o}{)}_{\mathrm{v}\mathrm{H}}$ and $\:(T\triangle{S}^{o}{)}_{\mathrm{v}\mathrm{H}}$ contributions. An alternative approach entails the study of the interaction between protein and a series of closely related ligands at a single temperature to detect what has been termed the compensation phenomenon [25]. Indeed, this approach was adopted in the first two demonstrations of extreme enthalpy–entropy compensation in protein-ligand interactions [1, 5].

### Interaction of Cyanide with Methemoglobins A and C

**Fig. 5:**
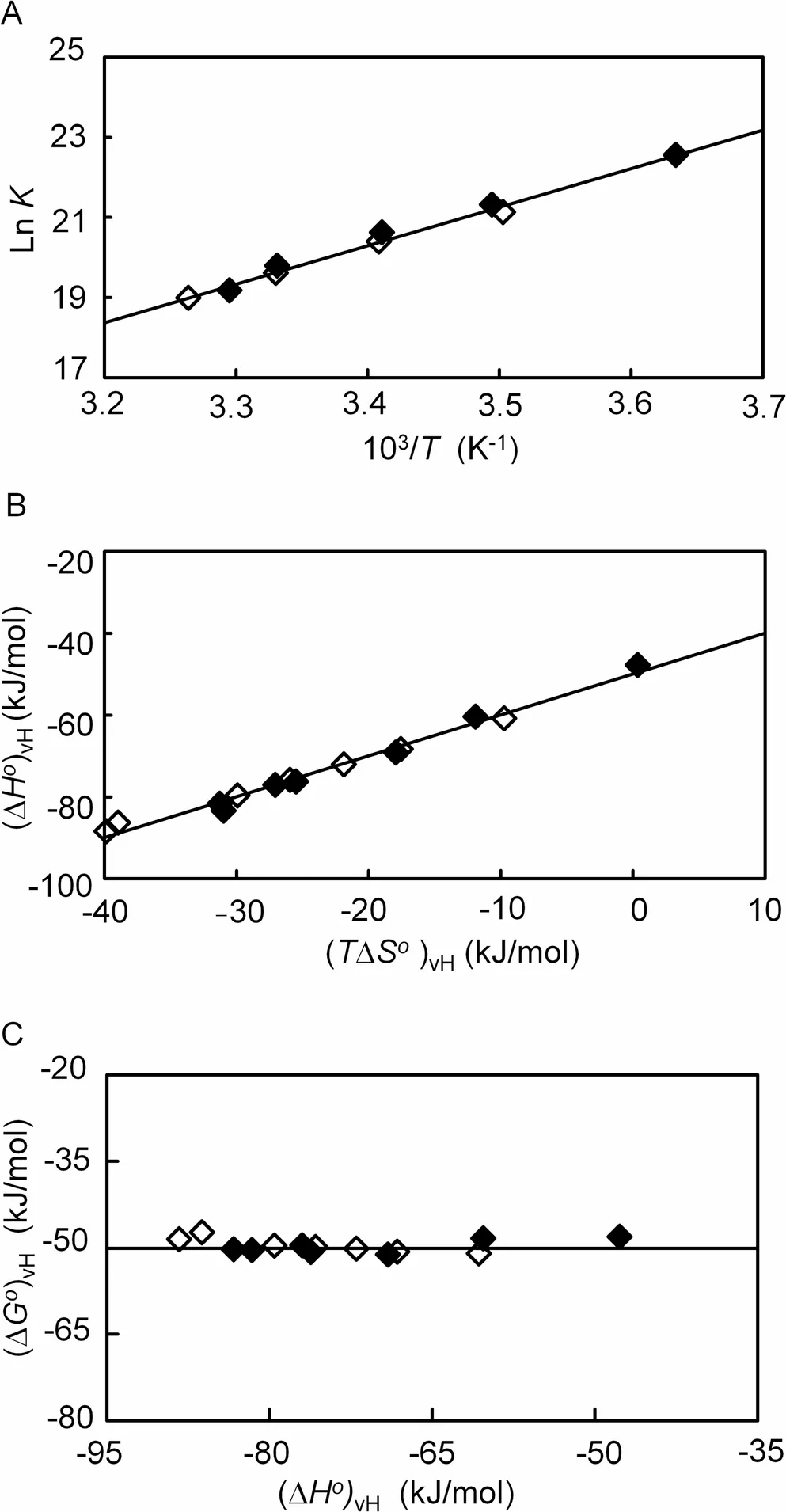
Demonstration of extreme enthalpy–entropy compensation from studies at a single temperature. **A** van’t Hoff analysis of spectral results on the basis of solvent inertness for the interaction of cyanide with methemoglobins A (◆) and C (◇) at 20 °C under a range of pH conditions. **B** Corresponding Gibbs–Helmholtz plot of the same results. **C** Temperature independence of (Δ $\:{G}^{o}{)}_{\mathrm{v}\mathrm{H}}$ upon (Δ*H*^*o*^)_cal_. (Data taken from Tables 13 and 14 of [1].)

In this investigation [1], the authors conducted spectral studies of the temperature dependence of cyanide interaction with the two methemoglobin variants to establish their conformity with Eq. (2) within the investigation [1], the most comprehensive summary of those spectral studies (Tables 13 and 14 therein) report $\:(\triangle{G}^{o}{)}_{\mathrm{v}\mathrm{H}}$, $\:(\triangle{H}^{o}{)}_{\mathrm{v}\mathrm{H}}\:$and $\:(T \triangle{S}^{o}{)}_{\mathrm{v}\mathrm{H}}$ values at 20 °C for a range of conditions. Analysis of those spectral results according to Eq. (2) are presented in Fig. 5A. Because that analysis is based on Eqs. (1)–(3), all of which are based on solvent inertness, their depiction as a Gibbs–Helmholtz plot necessarily has a slope indistinguishable from unity (Fig. 5B). The existence of solvent involvement was inferred [1] from the independence of $\:(\triangle{G}^{o}{)}_{\mathrm{v}\mathrm{H}}$ upon $\:(\triangle{H}^{o}{)}_{\mathrm{v}\mathrm{H}}$ (see Fig. 5C).    

That inferred evidence of extreme enthalpy–entropy compensation is consistent with the involvement of solvent (water) in the binding of cyanide to the sixth coordination position of the ferric ion in a heme ring structure that is embedded in a highly hydrophobic environment generated by the surrounding polypeptide chain. While these findings confirmed the Kauzman concept [24] of a positive entropy contribution arising from water involvement in hydrophobic interactions, they also drew attention to the fact that it has no impact on the magnitude of Δ*G*^*o*^ because any enthalpic advantage from the strengthening of water hydrogen bonding is automatically offset by the consequent decrease in randomness of the water structure [1, 12, 26].

Similar conformity of $\:(\triangle{G}^{o}{)}_{\mathrm{v}\mathrm{H}}$, $\:(\triangle{H}^{o}{)}_{\mathrm{v}\mathrm{H}}\:$and $\:(T\triangle{S}^{o}{)}_{\mathrm{v}\mathrm{H}}$with the requirements of the Gibbs–Helmholtz equation was observed in a subsequent competitive binding study of the interaction between agonists and antagonists with dopamine D-2 receptors at 4 °C [5]. Indeed, this was an investigation that popularized the concept that hydrophobic interactions make no contribution to the standard free energy change.

### Interaction of Charged Tripeptides with the Oligopeptide Binding Protein OppA

The binding of charged tripeptides (KXK) to OppA affords a system in which electrostatic attraction is the dominant interaction even though the tripeptide is completely buried in the protein interior [3]. In this study isothermal titration calorimetry was used to quantify the OppA interaction with a range of related ligands to effect changes in (Δ*H*^*o*^)_cal_ at single temperature (25 °C). An unusual feature to emerge from the isothermal titration calorimetry study [3] is the consistent return of positive values for $\:(\triangle{H}^{o}{)}_{\mathrm{c}\mathrm{a}\mathrm{l}}$, which signifies that all tripeptide interactions are entropically driven to render $\: \triangle{G}^{o}\:$negative. As noted previously [27], OppA exhibits significantly weaker affinity for six of the twenty KXK tripeptides tested, which therefore form a separate subset for analysis. The two subsets have been normalized [28] by expressing $\:(\triangle{G}^{o}{)}_{\mathrm{v}\mathrm{H}}$ for each system as $\:(\triangle{G}^{o}{)}_{\mathrm{v}\mathrm{H}},$ its difference from the mean value of $\:(\triangle{G}^{o}{)}_{\mathrm{v}\mathrm{H}}$ for a given subset: the same procedure was used to calculate (ΔΔ*H*^*o*^ )_cal_.

**Fig. 6:**
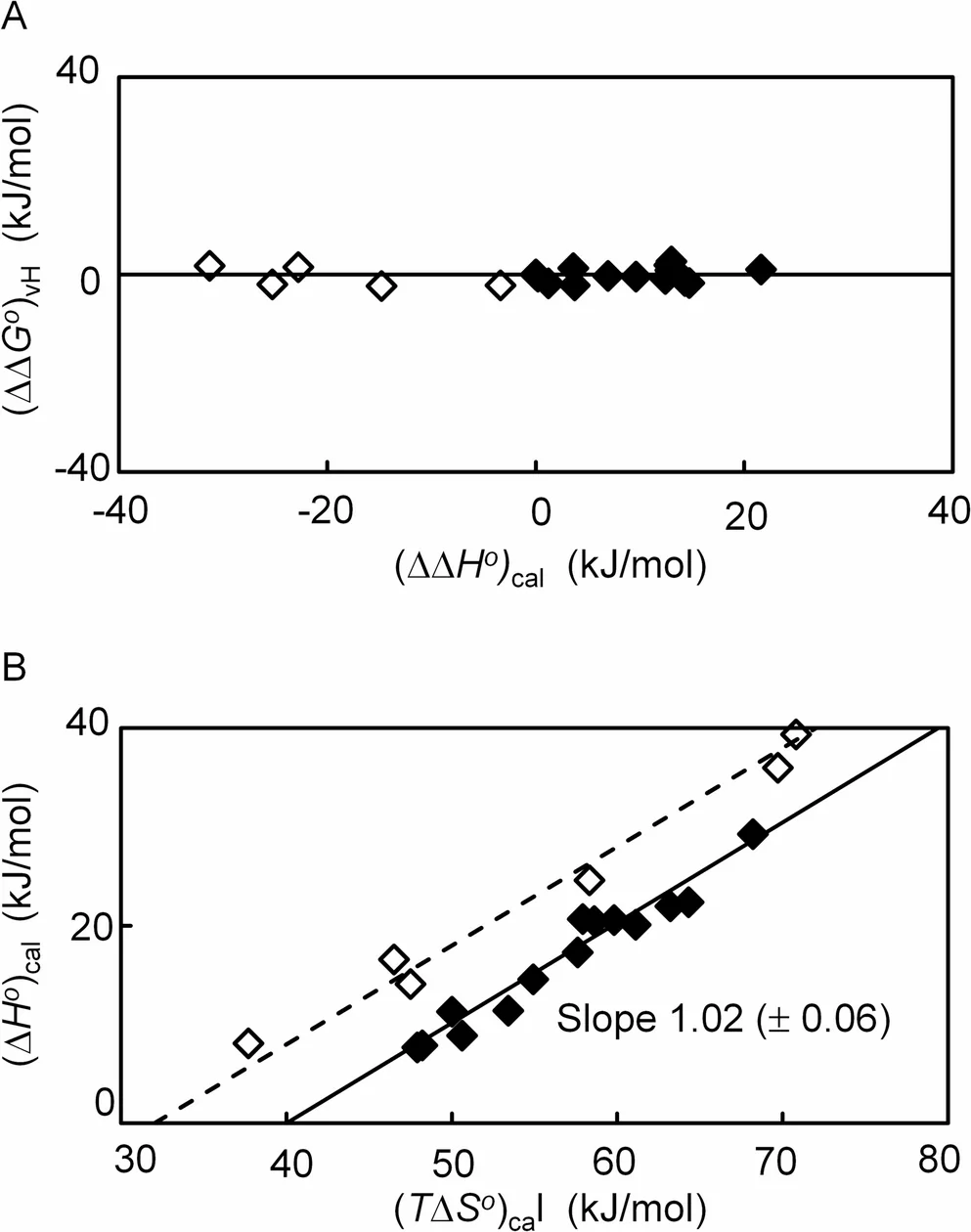
Evidence of extreme enthalpy–entropy compensation from a calorimetric study of the interaction between OppA and a range of KXK tripeptides at 25 °C. **A** Independence of the measured standard free energy change, (Δ$\:{G}^{o}{)}_{\mathrm{v}\mathrm{H}}$ upon (Δ*H*^*o*^)_cal_ for the major (◆) and minor (◇) data subsets. **B** Gibbs–Helmholtz plots of the separate data sets. (Data taken from Table 1 of [3].)

The resulting independence of $\:(\triangle{G}^{o}{)}_{\mathrm{v}\mathrm{H}}$ upon (ΔΔ*H*^*o*^)_cal_, shown in Fig. 6A, is consistent with a small magnitude for (Δ*H*^*o*^)_vH_ in both subsets; and hence a slope of essentially unity for the two Gibbs–Helmholtz plots (Fig. 6B). As required, the ordinate intercept, − 41.0 (± 3.9) kJ/mol, of the plot for the larger subset is indistinguishable from the mean value, − 40.3 kJ/mol, of $\:(\triangle{G}^{o}{)}_{\mathrm{v}\mathrm{H}}$ measurements used for the calculation of $\:(\triangle{G}^{o}{)}_{\mathrm{v}\mathrm{H}}$: the broken line through the smaller subset describes the corresponding relationship based on its mean value of − 32.1 kJ/mol for $\:(\triangle{G}^{o}{)}_{\mathrm{v}\mathrm{H}}$ and a slope of unity.

In summary, the solvent-mediated energy contribution is contained within $\:(\triangle{G}^{o}{)}_{\mathrm{v}\mathrm{H}}$, whereupon $\:(T\triangle{S}^{o}{)}_{\mathrm{c}\mathrm{a}\mathrm{l}}$ equates with $\:(T\triangle{S}^{o}$)_vH_ and $\:(\triangle{H}^{o}{)}_{\mathrm{c}\mathrm{a}\mathrm{l}}$ with $\:(\triangle{H}^{o}$)_vH_ in the standard analysis based on solvent inertness. For this system its source has been traced to the existence of structured water molecules entrapped in the highly hydrophobic binding site region of the OppA-tripeptide complex [3, 29].

### Interaction of Purine Nucleoside Phosphorylase with Transition State Analogues

**Fig. 7:**
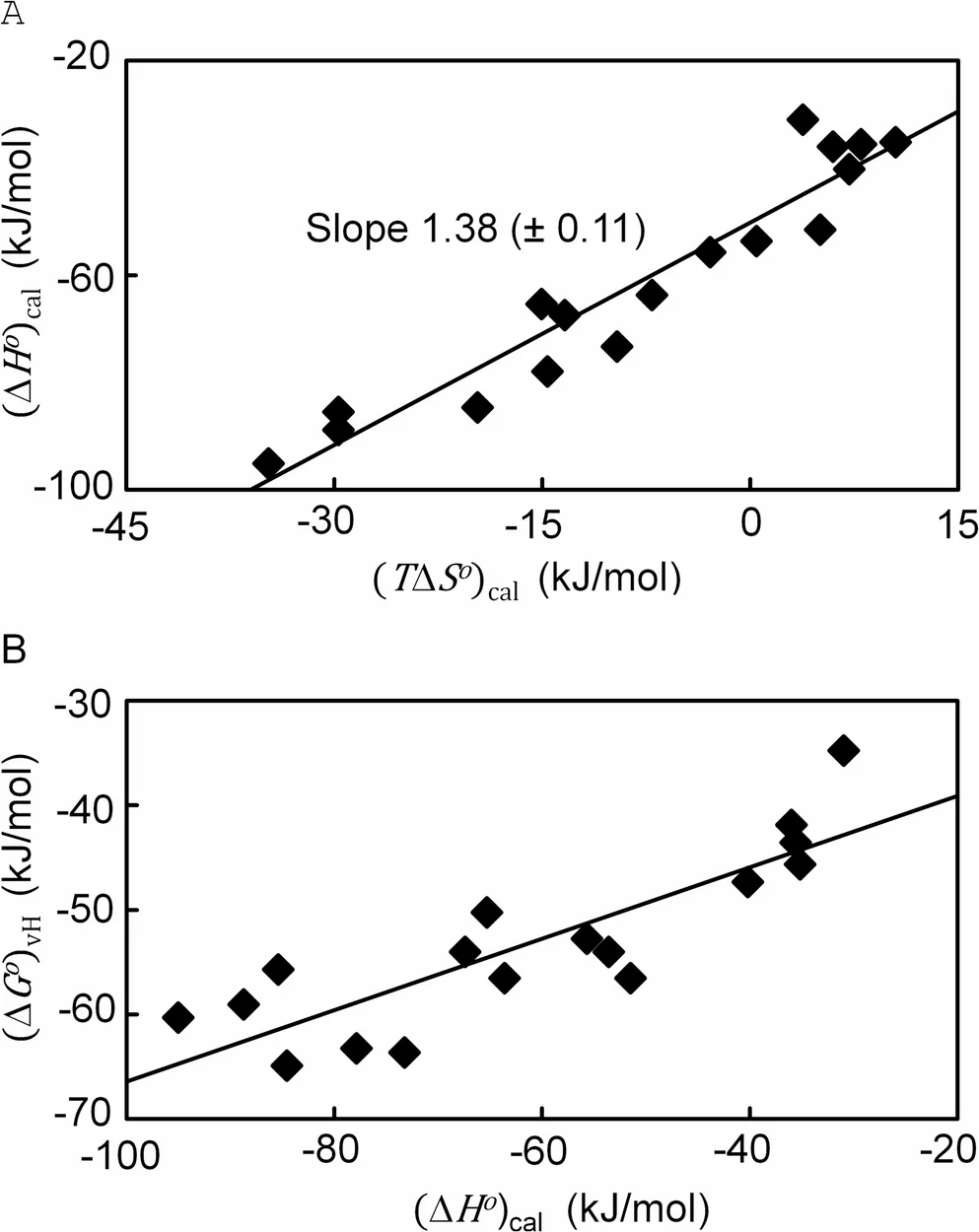
Calorimetric study of the interaction of purine nucleoside phosphorylase with a range of transition state analogues at 25 °C. **A** Dependence of (Δ*H*^*o*^)_cal_ upon (*T*Δ*S*^*o*^)_cal_ revealing nonconformity with the Gibbs–Helmholtz equation. **B** Dependence of the standard free energy change, (Δ$\:{G}^{o}{)}_{\mathrm{v}\mathrm{H}},$ upon (Δ*H*^*o*^)_cal_ as a potential source of that disparity. (Data taken from Table 1 of [10]

In an investigation of ligand binding to purine nucleoside phosphorylase [10] the existence of enthalpy–entropy compensation was also deduced from binding and calorimetric measurements at a single temperature (25 °C) with a range of related ligands to effect changes in (Δ*H*^*o*^)_cal_ For this system, however, the slope of 1.38 for the dependence of (Δ*H*^*o*^)_cal_ upon $\:(T \triangle{S}^{o})$)_cal_ (Fig. 7A) is significantly higher than the value of unity predicted by the Gibbs–Helmholtz equation for an inert solvent system.

A similar situation was encountered in calorimetric studies of the temperature dependence of the energetics for DppA-dipeptide interactions (Fig. 4C). For that system the larger than predicted slope of the Gibbs–Helmholtz plot was traced to deviation from the independence of standard free energy change upon its enthalpic counterpart that is associated with extreme enthalpy–entropy compensation. That situation also applies to the purine nucleoside phosphorylase interactions with transition analogues (Fig. 7B). These two systems should probably by regarded as examples of substantial rather than extreme enthalpy–entropy compensation.

## Discussion

The Gibbs–Helmholtz equation has traditionally provided the means of resolving the standard free energy change for a protein-ligand interaction into its enthalpic and entropic contributions. However, the validity of its reliance upon assumed inertness of the solvent came into question in the 1960s with the realization that hydrophobic interactions were a consequence of water reactivity arising from its hydrogen bonding capability [24, 30]. The reluctance to diverge from the concept of solvent inertness is evident from a much later calorimetric study by the Sturtevant group [19] of the interaction between ribonuclease A and a substrate analogue. Only after a failed attempt to account for the disparity between calorimetric measurements of (Δ*H*^*o*^)cal and estimates of the van’t Hoff parameter, (Δ*H*^*o*^)_vH_, incorporating a Δ*C*_*p*_ allowance (Figs. 1A, B) was the concession made that solvent must in some way be contributing to the energetics of the interaction. Reanalysis of those results (Figs. 2A, B) established their conformity with behavior commensurate with that for water involvement in the interaction. Solvent involvement has also been invoked as the source of enthalpy–entropy compensation in DNA-protein interactions [4, 31–37].

As well as examining studies in which temperature variation has been used to expose solvent-mediated enthalpy–entropy compensation, we have also considered situations where existence of the phenomenon has been established from studies of protein-ligand interactions with a range of ligands at a single temperature (Figs. 5 and 6). Analyses of results for the interactions of DppA and the dipeptide FA (Fig. 4) and of purine nucleoside phosphorylase (Fig. 7) provide a case for their reclassification as systems exhibiting substantial rather than extreme enthalpy–entropy compensation.

In summary, this retrospective but by no means comprehensive review of enthalpy–entropy compensation has followed developments in the field since its inception. Initial reports of extreme enthalpy–entropy compensation in protein-ligand interactions elicited expressions of amazement as well as disbelief. Consideration of such findings to be remarkable [38] and paradoxical [39] also led to their dismissal as statistical artifacts [40–42]. Inasmuch as the original experimental quantification of the entropic energetics contribution $\:(T\triangle{S}^{o}{)}_{\mathrm{v}\mathrm{H}}\:$entailed its evaluation as the difference between $\:(\triangle{H}^{o}{)}_{\mathrm{v}\mathrm{H}}$ and$\:\:(\triangle{G}^{o}{)}_{\mathrm{v}\mathrm{H}}$, no chemical significance could be accorded the slope of unity for a $\:(\triangle{H}^{o}{)}_{\mathrm{v}\mathrm{H}}\:versus\:T(\triangle{S}^{o}{)}_{\mathrm{v}\mathrm{H}}$when the magnitudes of $\:(\triangle{H}^{o}{)}_{\mathrm{v}\mathrm{H}}$ and $\:(\triangle{G}^{o}{)}_{\mathrm{v}\mathrm{H}}$, are interdependent because of the evaluation of the former by incorporating the experimental value of $\:(\triangle{G}^{o}{)}_{\mathrm{v}\mathrm{H}}$ the Gibbs–Helmholtz equation. Fortunately, that situation is now obviated by obtaining an independent estimate of the standard enthalpy energy contribution by isothermal titration calorimetry.

There is now widespread support for the existence of solvation effects in aqueous protein-ligand interactions [12–15, 42–45]: indeed, its potential role in surface chemistry is now also under scrutiny [46]. On the other hand, the adoption of a quantum-mechanical approach has led to the conclusion that the compensation phenomenon occurs to some extent, regardless of solvent effects in thermodynamic binding studies in the gas phase [47–49]—a reflection of the incorporation of the decreased randomness associated with complex formation between two molecules (the entropy compensation included in the original Gibbs–Helmholtz equation). Of interest in that regard is the use of results from a quantum mechanical investigation of the adverse effect of water on urea dimerization [50] to justify interpreting the thermodynamic observation of extreme enthalpy–entropy compensation in terms of solvent involvement in the urea-urea interaction [51]. However, the enhanced enthalpic contribution in that system reflected the strengthening of water hydroxyl interactions through competition for the urea carbonyl—a solvent-solute interaction that is incorporated readily into molecular dynamic simulations.

A unique feature of enthalpy–entropy compensation in many calorimetric studies of protein-ligand interactions is the observation of an enthalpic contribution that increases with increasing temperature—behaviour seemingly at odds with the exothermic nature of hydrogen bonding. However, it is compatible with the behavior of water molecules merely entrapped within the hydrophobic environment generated by the protein-ligand interaction. As noted by Kauzman [24], water structure is strengthened in a hydrophobic environment; and there is more scope for its strengthening is enhanced by temperature increase. At 278 K, water structure is essentially ice-like, and hence the entrapment of water molecules with a ligand-generated hydrophobic environment leads to only a minor enthalpic contribution to the energetics of the protein-ligand interaction. At higher temperatures water exists in a less-structured state, whereupon that extent of enthalpic energetic enhancement should, indeed, increase with increasing temperature despite its source as a change in extent of hydrogen bonding. At present this relatively simple thermodynamic explanation of enthalpy–entropy compensation in protein-ligand interactions seems to have escaped detection in molecular dynamic simulation.

Finally, our molecular understanding of enthalpy–entropy compensation is still in its relative infancy; but as Lewis Randall [52] noted in the preface to their monumental 1923 dissertation, “*The fascination of a growing science lies in the work of the frontiers at the very borderland of the unknown*,* but to reach this frontier one must pass over the well travelled roads; of these one of the safest and surest is the broad highway of thermodynamics”.* This piece of philosophy, revolutionary at that time, retains relevance today.

## Data Availability

No datasets were generated or analysed during the current study.

## References

[1] Anusiem AC, Beetlestone DH, Irvine DH (1968) Reactivity differences between haemoglobins. VI. The thermodynamics of the reactions of human methaemoglobins A and C with fluoride, thiocyanate and cyanide ions: an interpretation of enthalpy changes in terms of hydration. J Chem Soc A 960–969

[2] Waksman G, Shoelson SE, Pant N, Cowburn D, Kuriyan J (1993) Binding of a high affinity phosphotyrosyl peptide to the srs SH2 domain: crystal structures of the complexed and peptide-free forms. Cell 72:777–790 10.1016/0092-8674(93)90405-f 7680960

[3] Sleigh SH, Seavers PH, Wilkinson AJ, Ladbury JE, Tame JRH (1999) Crystallographic and calorimetric analyses of peptide binding to OPPA protein. J Mol Biol 191:393–415 10.1006/jmbi.1999.2929 10438628

[4] Dragan AJ, Read CM, Makeyeva EN, Miltogina EL, Churchill ME, Crane-Robinson C, Privalov PL (2004) DNA binding and bending by HMG boxes: energetic determination of specificity. J Mol Biol 343:371–393 10.1016/j.jmb.2004.08.035 15451667

[5] Kilpatrick JG, El Tayar N, Van de Waterbeemd H, Jenner P, Testa B, Marsden CD (1986) The thermodynamics of agonist and antagonist binding in dopamine D-2 receptors. Mol Pharmacol 30:226–234 2943980

[6] Krishnamurthy VM, Bohall BR, Semetey V, Whitesides GM (2006) The paradoxical thermodynamic basis for the attraction of ethylene glycol, glycine and sarcosine chains with carbonic anhydrase. II. An unexpected manifestation of enthalpy/entropy compensation. J Am Chem Soc 128:5802–5812 10.1021/ja060070r PMC2518651 16637649

[7] Lafont V, Armstrong AA, Ohtaka H, Kiso Y, Mario Amsel L, Freire E (2007) Compensating enthalpy and entropy changes hinder binding affinity optimization. Chem Biol Drug Discov 69:413–422 10.1111/j.1747-0285.2007.00519.x 17581235

[8] Williams DH, O’Brien DP, Sandercock AM, Stephens E (2004) Order changes within acceptor systems upon ligand binding: binding parameters. J Mol Biol 340:373–383 10.1016/j.jmb.2004.04.056 15201058

[9] Frederick KK, Ma MS, Valentine KG, Wand AJ (2007) Conformational entropy in molecular recognition by proteins. Nature 448:325–329 10.1038/nature05959 PMC4156320 17637663

[10] Edwards AA, Anusiem AC, Clinch K, Tylor PC, Evans GB, Schramm VL (2009) Altered enthalpy-entropy compensation in picomolar transition state analysis of human purine nucleotide phosphorylases. Biochemistry 48:5226–5238 10.1021/bi9005896 PMC2711852 19425594

[11] Ferranti A, Gorsky J (2012) Enthalpy-entropy compensation and cooperativity as thermodynamic epiphenomena in ligand-receptor interactions. J Mol Biol 417:454–467 10.1016/j.jmb.2012.01.057 PMC3349339 22342886

[12] Lumry R, Rajender S (1970) Enthalpy-entropy compensation phenomena in water solutions of proteins and small molecules: a ubiquitous property of water. Biopolymers 9:1125–1227 10.1002/bip.1970.360091002 4918636

[13] Clothia C (1974) Hydrophobic bonding and accessible surface area in proteins. Nature 248:338–339 10.1038/248338a0 4819639

[14] Reynolds JA, Gilbert DB, Tanford C (1974) Empirical correlation between hydrophobic free energy and aqueous cavity surface area. Proc Natl Acad Sci USA 71:2925–2927 10.1073/pnas.71.8.2925 PMC388590 16578715

[15] Breiten B, Lockett MR, Sherman W, Fujita S, Al-Sayah M, Lange H, Bower CH, Geroux A, Kilov G, Whitesides GM (2013) Water networks contribute to enthalpy-entropy compensation in protein-ligand binding. J Am Chem Soc 135:15579–15584 10.1021/ja4075776 24044696

[16] Klotz IM, Franzen JS (1962) Hydrogen bonds between model peptides in solution. J Am Chem Soc 84:3461–3466

[17] Schrier EE, Pottle M, Scheraga HA (1964) The influence of hydrogen and hydrophobic bonds on the stability of the carboxylic acid dimers in aqueous solution. J Am Chem Soc 86:3444–3449

[18] Wiseman T, Williston S, Brandts JF, Lin L-N (1989) Rapid measurements of binding constants and heats of binding using a new titration calorimeter. Anal Biochem 179:131–137 10.1016/0003-2697(89)90213-3 2757186

[19] Naghibi H, Tamura A, Sturtevant JM (1995) Significant discrepancies between van’t Hoff and calorimetric enthalpies. Proc Natl Acad Sci USA 92:5597–5599 10.1073/pnas.92.12.5597 PMC41743 7777555

[20] Weber G (1995) van’t Hoff revisited: enthalpy of association of protein subunits. J Phys Chem 99:1052–1059

[21] Leonidas DD, Chavali GB, Oikonomakos NG, Chrysina ED, Kosmopoulou MN, Vlassi M, Frankling C, Ravi Acharya K (2003) High-resolution structure of ribonuclease A complexed with adenylic and uridylic nucleotide inhibitors: implications for structure-based design of ribonucleolytic inhibitors. Protein Sci 12:2559–2574 10.1110/ps.03196603 PMC2366950 14573867

[22] Zainol MKM, Linforth RJC, Winzor DJ, Scott DJ (2021) Thermodynamics of semi-specific ligand recognition: the binding of dipeptides to the *E. coli* dipeptide binding protein DppA. Eur Biophys J 50:1103–1110 10.1007/s00249-021-01572-y PMC8566422 34611772

[23] Dunten P, Mowbray SL (1995) Crystal structure of the dipeptide binding protein from *Escherichia coli* involved in active transport of and chemotaxis. Protein Sci 4:1322–2234 10.1002/pro.5560041110 PMC2143009 8563629

[24] Kauzman W (1959) Some factors in the interpretation of protein denaturation. Adv Protein Chem 14:1–63 10.1016/s0065-3233(08)60608-7 14404936

[25] Liu L, Guo Q-X (2001) Isokinetic relationship, isoequilibrium relationship, an enthalpy-entropy compensation. Chem Rev 101:673–695 10.1021/cr990416z 11712500

[26] Ives DJC, Marsden PD (1965) The ionisation functions of di-isopropylcyanoacetic acid in relation to hydration equilibria and the compensation law. J Chem Soc 649–676

[27] Scott DJ, Tame JRH, Winzor DJ (2019) Rationalization of enthalpy-entropy compensation in terms of protein conformational entropy and solvent structure perturbation. Curr Top Biochem Res 19:59–69

[28] Olsson TGA, Ladbury JE, Pitt WR, Williams MA (2011) Extent of enthalpy-entropy compendsation in protein-ligand interactions. Protein Sci 20:1608–1618 10.1002/pro.692 PMC3190155 21739503

[29] Tame RJH, Murshadov GN, Dodson EJ, Neil K, Dodson GG, Higgins CF, Wilkinson AJ (1994) The structural basis of sequence-independent binding by OppA protein. Science 264:1578–1158 10.1126/science.8202710 8202710

[30] Tanford C (1981) The hydrophobic effect. Wiley, New York

[31] Jen-Jacobson L, Engler LE, Jen-Jacobson LA (2000) Structural and thermodynamic strategies for site-specific DNA binding proteins. Structure 8:1015–1023 10.1016/s0969-2126(00)00501-3 11080623

[32] Dragan AI, Klass J, Read C, Churchill MEA, Crane-Robinson C, Privalov PL (2003) DNA binding of a non-sequence-specific HMG-D protein is entropy riven with a substantial non-electrostatic contribution. J Mol Biol 331:795–813 10.1016/s0022-2836(03)00785-x 12909011

[33] Dragan AI, Franks L, Liu Y, Makeyeva EN, Crane-Robinson C, Privalov PL (2004) Thermodynamic signature of GCN4-bZIP binding to DNA indicates the role of water in discriminating between the AP-1 and ATF/CREB sites. J Mol Biol 343:865–878 10.1016/j.jmb.2004.08.101 15476806

[34] Dragan AI, Li Z, Makeyeva EN, Milgotina EL, Liu Y, Crane-Robinson C, Privalov PL (2006) Forces driving the homeodomains to DNA. Biochemistry 45:141–151 10.1021/bi051705m 16388589

[35] Privalov PL, Dragan AI, Crane-Robinson C, Breslauer KJ, Remeter DF, Minetta SA (2007) What drives proteins into the major or minor grooves of DNA? J Mol Biol 365:1–9 10.1016/j.jmb.2006.09.059 PMC1934558 17055530

[36] Privalov PL, Crane-Robinson C (2017) Role of water in the formation of macromolecular structures. Eur Biophys J 46:203–224 10.1007/s00249-016-1161-y PMC5346440 27457765

[37] Dragan AI, Read CM, Crane-Robinson C (2017) Enthalpy-entropy compensation: the role of solvation. Eur Biophys J 46:303–308 10.1007/s00249-016-1182-6 PMC5384952 27796417

[38] Gilli P, Ferretti V, Borea TA (1994) Enthalpy-entropy compensation in drug-receptor binding. J Phys Chem 98:1515–1518

[39] Krishnamurthy VM, Bohall BR, Semetey V, Whitesides GM (2006) The paradoxical thermodynamic basis for the attraction of ethylene glycol, glycine and sarcosine chains with carbonic anhydrase: II. An unexpected manifestation of enthalpy/entropy compensation. J Am Chem Soc 128:5902–5812 10.1021/ja060070r PMC2518651 16637649

[40] Sharp K (2001) Enthalpy-entropy compensation: fact or artifact. Protein Sci 10:661–667 10.1110/ps.37801 PMC2374136 11344335

[41] Cornish-Bowden A (2002) Enthalpy-entropy compensation: a phantom phenomenon. J Biosci 27:121–126 10.1007/BF02703768 11937682

[42] Chodera JD, Mobley DL (2013) Enthalpy-entropy compensation: role and ramifications in biomolecular recognition and design. Annu Rev Biophys 42:121–114 10.1146/annurev-biophys-083012-130318 PMC4124006 23654303

[43] Yu HA, Karplus M (1988) A thermodynamic analysis of solvation. J Chem Phys 89:2366–2388

[44] Grunwald E, Steel C (1995) Solvent reorganization and thermodynamic enthalpy-entropy compensation. J Am Chem Soc 117:5687–5692

[45] Mancini F, Graziano G (2025) On enthalpy-entropy compensation characterization processes. Entropy 27:716 10.3390/e27070716 PMC12295146 40724433

[46] Pethica BA, Mingins J (2026) Gibbs adsorption isotherm: the relevance of solvent in the adsorption layer. Langmuir 42:142–145 10.1021/acs.langmuir.5c03262 41432921

[47] Korth M (2013) A quantum chemical view of enthalpy-entropy compensation. MedChemComm 4:1025–1033

[48] Ryde U (2014) A fundamental view of enthalpy-entropy compensation. MedChemComm 5:1324–1325

[49] Sokolov AA, Khisamiev MB, Solomonov BN, Yagofarov MI (2025) Phys Chem Chem Phys 27:26159 10.1039/d5cp04241k 41307277

[50] Ramondo F, Bencivenni R, Caminiti A, Pieretti L, Gontrani L (2007) Dimerization of urea in water solution: a quantum mechanical investigation. Phys Chem Chem Phys 2206–2216 10.1039/b617837e 17487317

[51] Winzor DJ, Wills PR (2019) Quantitative interpretation of isopiestic measurements on aqueous urea solutions: urea revisited. Biophys Chem 251:106175 10.1016/j.bpc.2019.106175 31128561

[52] Lewis GN, Randall M (1923) Thermodynamics and the free energy of chemical substances. McGraw-Hill, New York

